# Scarcity mindset facilitates empathy for social pain and prosocial intention: behavioral and neural evidences

**DOI:** 10.1093/scan/nsaf015

**Published:** 2025-01-28

**Authors:** Wanchen Li, Zilong Wei, Jun Wu, Ru Song, Jie Liu, Fang Cui

**Affiliations:** School of Psychology, Shenzhen University, Shenzhen 518060, China; School of Psychology, Shenzhen University, Shenzhen 518060, China; School of Psychology, Shenzhen University, Shenzhen 518060, China; School of Psychology, Shenzhen University, Shenzhen 518060, China; School of Psychology, Shenzhen University, Shenzhen 518060, China; Center for Brain Disorders and Cognitive Neuroscience, Shenzhen University, Shenzhen 518060, China; School of Psychology, Shenzhen University, Shenzhen 518060, China; Center for Brain Disorders and Cognitive Neuroscience, Shenzhen University, Shenzhen 518060, China

**Keywords:** scarcity mindset, empathy for social pain, prosocial intention, event-related potential, late positive potential (LPP)

## Abstract

Empathy for social pain encompasses both affective and cognitive responses to others’ emotional reactions following negative social encounters, facilitating an understanding of their suffering and promoting prosocial behaviors. This study examined how a scarcity mindset affects empathy for social pain and prosocial intentions at behavioral and neural levels. Sixty participants were randomly assigned to either the scarcity or abundance mindset group. They viewed images of social exclusion or neutral scenarios and subsequently rated the perceived unpleasantness of the target person and their willingness to provide comfort during a stage-game paradigm. The results showed that participants in the scarcity mindset group demonstrated greater differentiation in their ratings of unpleasantness and willingness to comfort when exposed to social exclusion images compared to neutral ones, relative to the abundance mindset group. Electrophysiological data revealed that social exclusion images elicited larger late positive potential (LPP) amplitudes in the scarcity mindset group, but not in the abundance mindset group. Additionally, within the scarcity mindset group, affective empathy trait scores moderated the relationship between LPP amplitudes and willingness to comfort ratings. These findings highlight the amplifying effects of a scarcity mindset on empathy for social pain and prosocial intentions, and emphasize the role of affective empathy traits in this dynamic process.

## Introduction

Empathy, often observed in response to negative physical or social experiences of others (Meyer et al. 2013, Wei et al. 2023), is essential for fostering social connections (Singer and Klimecki 2014). It is a multifaceted concept (Cuff et al. 2016) that comprises three key components (Zaki and Ochsner 2012): affective empathy, which involves sharing and indirectly experiencing the emotional states of others (Gallese et al. 2004, Decety and Cowell 2014, Marsh 2018); cognitive empathy, the ability to accurately understand how individuals perceive and experience their situations (Leslie et al. 2004, Decety and Cowell 2014, Marsh 2018, Blanke and Riediger 2019); and prosocial disposition, which reflects the motivation to support the well-being of others or to help alleviate their suffering (Zaki and Ochsner 2012).

Empathy encompasses the ability to understand and share not only others’ primary emotions (Krach et al. 2015), such as physical pain (Singer et al. 2004), but also more complex social emotions including social rejection and embarrassment (Krach et al. 2011, Paulus et al. 2015). Social pain, a specific subtype of emotional pain (Levi et al. 2023), is characterized by intense emotional distress triggered by adverse social experiences, such as social exclusion (Macdonald and Leary 2005). Witnessing instances of social exclusion can lead individuals to vicariously experience associated emotional pain, a phenomenon known as empathy for social pain. This allows them to emotionally resonate with and cognitively comprehend the distress of those who are socially rejected (Atkins et al. 2016).

Empathy is widely recognized as a catalyst for prosocial behavior (Decety et al. 2016). It enhances individuals’ ability to recognize the needs of others, motivating them to engage in prosocial behaviors and respond more readily to requests for assistance (Lockwood et al. 2016, Pang et al. 2022). The empathy-altruism hypothesis posits that empathy for someone in need or distress generates altruistic motivation, which subsequently fosters the desire to engage in prosocial behaviors (Batson et al. 1988, 2015). Supporting empirical studies have consistently demonstrated that feeling empathy significantly enhances individuals’ likelihood of engaging in prosocial behaviors directed toward the target of their empathy (Batson et al. 1981, 1999, 2002). When individuals empathize with the social pain of others, they are more likely to engage in various prosocial behaviors toward ostracized victims, such as sympathy, assistance, and comfort (Masten et al. 2010, 2011, Wesselmann et al. 2013). Extensive research has examined the impact of various social factors on empathic responses to others’ physical pain, as well as the underlying neural mechanisms involved. For instance, specific social contexts can influence neural responses when observing others’ pain. A threatening context enhances early neural reactions to others’ suffering, whereas a friendly context promotes later empathic processing (Cui et al. 2017). Additionally, empathic responses to the suffering of others are affected by one’s relative position in the social hierarchy; individuals tend to exhibit greater empathy toward those of inferior status than toward those of superior status (Feng et al. 2016). Moreover, observing a situation that may be painful for others can elicit feelings of empathy, even when the individuals involved are different from the observers and the situation does not cause any pain to the observers themselves (Perry et al. 2010). Furthermore, beliefs about others’ pain can modulate empathic responses, with empathy being diminished in the absence of such beliefs (Wu and Han 2021). However, research on empathy for social pain across different social contexts is relatively new and limited.

Resource scarcity poses a significant challenge globally, affecting not only the economically disadvantaged but also others. A scarcity mindset is often triggered by feelings of insufficient resources (Shah et al. 2012, Huijsmans et al. 2019). This mindset fosters a self-centered bias (Roux et al. 2015), leading individuals who perceive themselves as having inadequate resources to exhibit reduced empathy toward others’ physical pain (Li et al. 2023) and diminished prosocial intentions (Aarøe and Petersen 2013, Cui et al. 2022, 2023, Hasson et al. 2022, Agneman et al. 2023). However, some authors have reached contrasting conclusions, suggesting that a scarcity mindset can foster other-oriented behaviors (Ye and Mattila 2023), leading to enhanced empathic accuracy (Kraus et al. 2010) and increased prosocial intentions (Piff et al. 2010, Louie and Rieta 2018, Goldsmith et al. 2020, Laforet et al. 2024). These inconsistent findings regarding the impact of a scarcity mindset on prosocial intentions may arise from its varying effects on empathy for physical pain compared to empathy for social pain, given the crucial role that empathy plays in motivating prosocial behaviors.

In the context of resource scarcity, individuals may become acutely aware of their limited resources, often neglecting the physiological needs of others and hesitating to share what little they have. However, alternative perspectives suggest that those facing resource scarcity may seek security in other areas (Cannon et al. 2019), such as by developing a greater preference for cooperation to forge social connections in the face of perceived socio-psychological resource scarcity (Civai et al. 2022). Consequently, they may remain attuned to others’ social and emotional cues. For example, individuals from lower social classes, who possess fewer resources, tend to absorb more contextual information when observing others’ emotions (Kraus et al. 2009). This heightened sensitivity can lead to more accurate judgments of others’ social emotions and greater empathy compared to their upper-class counterparts (Kraus et al. 2010, 2012).

Empathy for social pain is likely to be a common and salient experience (Masten et al. 2010), given the inevitability of encountering social pain in interpersonal relationships and its enduring effects (Chen et al. 2008). Exploring how a scarcity mindset influences empathy for social pain and related prosocial intentions, such as comforting others, can enhance our understanding of individuals’ social behaviors during times of resource scarcity. Furthermore, this study provides insights into whether the effects of a scarcity mindset on empathy for social pain differ from previous findings related to empathy for physical pain.

In recent years, an increasing number of researchers have focused on temporal neural processing of empathy for social pain. Specifically, event-related potential (ERP) studies on adult college students have revealed a pronounced late positive potential (LPP) component during the observation of social exclusion images, indicating heightened emotional responses to social exclusion stimuli (He et al. 2020, Zhao et al. 2021). Additionally, other studies have shown that empathy for social pain in participants aged 18–50 years elicits both the early N2 component over fronto-central electrodes, which is associated with sharing others’ emotions, and the late P3 component over centro-parietal electrodes, which reflects prolonged attentional processing and cognitive appraisal of stimuli with motivational significance (Flasbeck and Brüne 2019, Wang et al. 2021, Rodriguez et al. 2023). A review of electrophysiological studies concluded that observing social exclusion is associated with N2, P3, and late slow-wave components, particularly in studies focusing on adults (Mills et al. 2024). Similar to empathy for physical pain (Fan and Han 2008), empathy for social pain involves both early bottom-up affective components, which reflect automatic emotional resonance with the emotional states of others, and later top-down processes related to attentional engagement and cognitive resource allocation. Building on previous research (Li et al. 2023), we hypothesized that a scarcity mindset may influence both the early bottom-up emotional resonance and the late top-down attentional processes involved in empathy for social pain.

To induce a scarcity mindset in a controlled laboratory setting, we employed a stage-game paradigm to manipulate scarcity and abundance mindsets (Huijsmans et al. 2019). In the scarcity condition, participants consistently faced the risk of not being able to advance, triggering a scarcity mindset. In contrast, participants in the abundance condition were able to proceed without stress, fostering an abundance mindset. This paradigm has been shown to effectively induce sustained feelings of scarcity (Huijsmans et al. 2019, Li et al. 2023). It was seamlessly integrated with the main task, thereby minimizing the likelihood of participant suspicion. Furthermore, the stimulus materials consisted of images depicting college students in various authentic campus social exclusion or neutral situations (Zheng et al. 2022). In this study, two distinct groups of participants viewed picture stimuli while experiencing either scarcity or abundance mindsets. We measured unpleasantness ratings and prosocial intention (i.e. willingness to comfort) ratings for the stimuli under these different conditions.

The present study aimed to investigate the effects of scarcity mindset on both behavioral and neural responses when individuals observe someone experiencing social exclusion. Building on prior literature suggesting that individuals facing resource scarcity exhibit heightened sensitivity to others’ social emotions (Kraus et al. 2010), we hypothesized that, unlike inhibiting empathy for physical pain, a scarcity mindset might amplify empathy for social pain and promote prosocial intentions. Specifically, at the behavioral level, we anticipated that participants in a scarcity mindset would report higher ratings of unpleasantness and a greater willingness to offer comfort when witnessing individuals subjected to social exclusion (as opposed to neutral) scenarios compared to those in an abundance mindset. At the neural level, we expected that the amplitudes of the early N2, as well as the later P3 and LPP components elicited by social exclusion (versus neutral) stimuli, would be greater under a scarcity mindset than under an abundance mindset.

## Materials and methods

### Participants

The sample size was determined using G*Power 3.1.9 software [Faul et al. 2009; *F*-test, repeated-measure analysis of variance (ANOVA), within-between interaction]. We conducted a priori power analysis based on a significant interaction effect on the LPP amplitudes observed in a previous ERP study (η_p_^2^ = 0.08; Li et al. 2023). The analysis indicated that the sample of 28 participants provided a statistical power of 95%. To further enhance the statistical power, we recruited 30 participants per group, resulting in a total of 60 participants (see Table 1). All participants were right-handed adults aged 18–25 years with normal or corrected-to-normal vision. The exclusion criteria included a history of neurological disorders, brain injuries, developmental disabilities, psychiatric disorders, or current use of psychoactive medications. These criteria were assessed using self-reporting questionnaires and interviews. Written informed consent was obtained from all the participants. The research protocol was approved by the Medical Ethics Committee of Shenzhen University, in accordance with all provisions outlined in the Declaration of Helsinki.

**Table 1.** Demographic and psychometric variables for two groups of participants (mean ± SD)

	Scarcity group	Abundance group	*P*
Demographic variables			
Age (years)	20.83 ± 2.07	20.87 ± 2.15	.951
Female/male	15/15	15/15	-
Psychometric variables			
Cognitive empathy	55.00 ± 8.12	55.03 ± 6.94	.986
Affective empathy	32.70 ± 5.07	31.77 ± 4.46	.452
Trait anxiety	40.80 ± 7.68	40.13 ± 8.77	.755
State anxiety	36.27 ± 6.94	36.47 ± 9.55	.926
Social anxiety	47.70 ± 19.59	52.50 ± 20.74	.361

Statistics were obtained using independent-sample *t*-tests.

Before the study, participants were asked to complete several assessments, including the State-Trait Anxiety Inventory (Spielberger et al. 1983), Liebowitz Social Anxiety Scale (Liebowitz 1987), and Questionnaire of Cognitive and Affective Empathy (Reniers et al. 2011). The levels of anxiety and empathy were comparable between the two groups. The participants completed these assessments prior to the main task, with a brief interval between the completion of these measurements and the start of the electroencephalography (EEG) experiment.

### Stimuli

**Figure 1.**
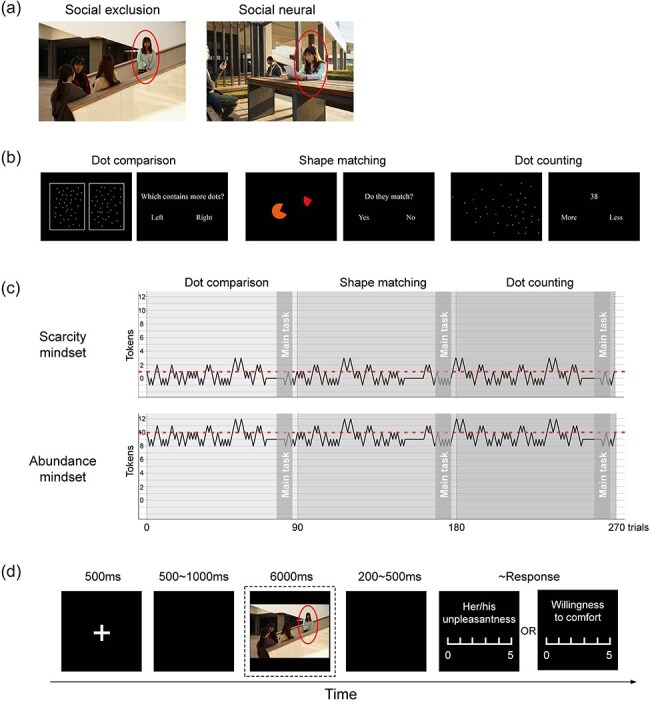
Experimental design. (a) Examples of social exclusion (left) and social neutral (right) pictures. (b) Illustrations of three stage-games. (c) A flowchart depicting the stage-game paradigm. The red dashed lines indicate the threshold, while the black lines represent the variation in the number of tokens around the threshold for each game under scarcity (top) and abundance (bottom) mindsets. The gray shaded areas highlight the main task stages. (d) A flowchart illustrating the main task. ERP responses were time-locked to the segment marked with a dashed-line box.

The images used in this study were sourced from the publicly accessible Image Database of Social Inclusion/Exclusion in Young Asian Adults (ISIEA; Zheng et al. 2022). Previous research has demonstrated that the ISIEA effectively presents negative social situations that evoke feelings of social distress in younger adults (Zheng et al. 2022, He et al. 2023, Mo et al. 2023, Yu et al. 2023). For this study, we selected 35 social exclusion images and 35 socially neutral images from the database, as illustrated in Fig. 1a. The social exclusion images depict scenarios in which one individual (the rejectee) displays sad or upset facial and body expressions, whereas three or four rejecters engage in conversation and laughter. In contrast, socially neutral images portray scenes featuring two to five individuals exhibiting neutral expressions, without any social interaction or communication. According to the ratings of physical parameters from the ISIEA (Zheng et al. 2022), significant differences were observed between the social exclusion and neutral images across various dimensions, including valence (3.33 ± 0.48 vs 5.08 ± 0.31, *P *< .001), arousal (4.61 ± 0.19 vs 4.22 ± 0.16, *P *< .001), inclusion score (3.22 ± 0.53 vs 5.31 ± 0.28, *P *< .001), and vicarious feeling (3.14 ± 0.36 vs 4.98 ± 0.30, *P *< .001). Each stimulus subtended a visual angle of 7.5°× 5.0° (width× height) with a viewing distance of 80 cm.

### Design and procedure

The present study utilized a 2 (Group: scarcity mindset, abundance mindset) × 2 (Picture: social exclusion, social neutral) mixed design. Each participant was randomly assigned to one of the mindset groups (scarcity or abundance), with allocation counterbalanced across participants. The experiment was conducted in a quiet, temperature-controlled room. Participants were instructed to focus on the experimental task displayed on a 24-inch computer screen using E-Prime 3.0 software (Psychology Software Tools, Inc., Pittsburgh, PA, USA). The EEG data were continuously recorded throughout the main task.

Scarcity and abundance mindsets were induced using an experimental manipulation known as three-stage games (Huijsmans et al. 2019). Each stage-game (dot comparison, shape matching, or dot counting; see Fig. 1b) consisted of a block of 90 trials. In this paradigm, the initial number of tokens was manipulated within the game, ensuring an equal number of wins and losses across identical stage games under both mindset conditions. To induce a scarcity mindset, participants were provided with one initial token, and the token count remained at approximately one throughout the study. In contrast, for the abundance mindset manipulation, participants received 10 initial tokens, with the token count fluctuating around 10 during the study (see Fig. 1c). Feedback on the outcome of each keypress was provided, and the current token count was displayed at the end of each stage. All participants were informed that they needed to have at least one token at the end of each game to receive a reward of 100 RMB; otherwise, they would only receive compensation of 10 RMB. A more detailed description of each stage-game can be found in Supplementary material S1.

The main task, including social exclusion stimuli, was intertwined with stage-games. During the interval of each stage-game, the participants engaged in the main task. In each trial of the main task (see Fig. 1d), a fixation cross was displayed on a black screen for 500 ms, followed by a random interval ranging from 500 to 1000 ms. Subsequently, either a social exclusion or neutral picture was presented for 6000 ms, during which participants were instructed to observe attentively. After a random interval of 200–500 ms, a 6-point numerical rating scale appeared. Participants were instructed to respond by pressing a key corresponding to a value from 0 to 5 to evaluate either “The level of unpleasantness of the target person, i.e. the one marked with a red circle (0 = no unpleasantness, 5 = enormous unpleasantness)” or “The level of your willingness to comfort the target person (0 = no willingness, 5 = enormous willingness).” The inter-trial interval varied randomly between 1000 and 2000 ms. Each block comprised 47, 46, and 47 trials, respectively.

Furthermore, to assess the effectiveness of scarcity and abundance mindset induction, participants were asked to provide subjective ratings of their stress, confidence, motivation, and excitement using 9-point Likert scales (see Supplementary material S2). Higher scores indicate greater levels of each rating. These assessments were collected at various time points, including the pre-experiment phase (baseline) and after the completion of each stage-game (posttest: T1, T2, T3).

### Electroencephalography data recording

EEG data were recorded from 64 scalp sites using tin electrodes mounted on an actiCHamp system (Brain Vision LLC, Morrisville, NC, USA; passband: 0.01–100 Hz; sampling rate: 1000 Hz). The FCz electrode was used as a recording reference, and the electrode on the medial frontal aspect was used as the ground electrode. All the electrode impedances remained below 5 kΩ.

### Electroencephalography data analysis

EEG data were preprocessed offline and analyzed using MATLAB R2019 (MathWorks, Natick, MA, USA) in conjunction with the EEGLAB toolbox (Delorme and Makeig 2004). Continuous EEG signals underwent band-pass filtering within the 0.1–30 Hz range. The signals were then segmented, starting 500 ms before stimulus onset and extending for 3000 ms, resulting in a total analysis window of 3500 ms. Baseline correction was applied using a 500-ms prestimulus time window. Epochs with amplitude values exceeding ±100 μV at any electrode were excluded from averaging. Additionally, EEG epochs were visually inspected, and trials containing significant noise due to gross movements were removed, accounting for 4.71% of the total number of epochs. Electrooculographic artifacts were corrected using an independent component analysis (ICA) algorithm (Jung et al. 2001). Following ICA and additional baseline correction, EEG trials were re-referenced to the bilateral mastoid electrodes.

For each participant, single-trial ERP waveforms elicited by social exclusion and social neutral pictures were averaged separately for scarcity and abundance mindset conditions. This process yielded four average waveforms per participant, each time-locked to the onset of the pictures for the respective mindset conditions. To obtain group-level ERP waveforms, single-participant average waveforms were averaged across all participants. Group-level scalp topographies were computed using spline interpolation. Based on the topographical distribution of the grand-averaged ERP activity and prior research (Zhao et al. 2021, Li et al. 2023), N2 amplitudes were measured at fronto-central electrodes (F1, Fz, F2, FC1, FCz, FC2) within the time window of 215–255 ms; P3 amplitudes were measured at parietal electrodes (CP3, CPz, CP4, P3, Pz, P4, PO3, POz, PO4) within the time window of 350–390 ms; and LPP amplitudes were measured at centro-parietal electrodes (C3, Cz, C4, CP3, CPz, CP4) within the time window of 500–3000 ms.

### Statistical analysis

For behavioral data, statistical analysis was conducted using the *lmerTest package* (Version 3.1.3; Kuznetsova et al., 2017) in R (Version 4.3.2). Linear mixed models (LMMs) were employed to evaluate ratings of unpleasantness and willingness to comfort. Group (scarcity mindset, abundance mindset) and picture types (social exclusion, social neutral) were defined as fixed effects, while participants and random intercepts were included as random effects to account for variability between participants. The effects of group and picture type on unpleasantness ratings and willingness to comfort were assessed separately. Descriptive statistics are presented as mean ± standard error (SE). Simple effect analyses were performed for any significant interaction. Statistical differences were considered significant at *P* < .05, with *P*-values corrected using the false discovery rate procedure.

For neural data and subjective ratings, statistical analyses were conducted using IBM SPSS Statistics 22 (IBM Corp., Armonk, NY, USA). Descriptive statistics are presented as mean ± SE. Neural responses (ERP components) were analyzed using two-way repeated-measures ANOVAs, including the between-participant factor of Group (scarcity mindset, abundance mindset) and the within-participant factor of Picture (social exclusion, social neutral). Differential subjective ratings (posttest–baseline) were analyzed using two-way repeated-measures ANOVAs, with Group (scarcity mindset, abundance mindset) and Timepoint (T1, T2, T3) as factors. In cases of significant interactions, post hoc pairwise comparisons were conducted, and significance levels (*P*-values) were Bonferroni-corrected at *P *< .05. The degrees of freedom for the F-ratios were adjusted using the Greenhouse–Geisser method.

To examine whether empathy traits moderated the relationship between neural and behavioral responses, a moderating effect analysis was conducted using the PROCESS macro in SPSS (Hayes 2012). In these moderating models, the independent variable (X) was the neural response (ERP amplitude), the dependent variable (Y) was the behavioral response (ratings of unpleasantness and willingness to comfort), and the moderator variable (Mo) was the score of empathy traits (affective and cognitive empathy). The conditioning values were set at −1SD, Mean, and +1SD in the PROCESS. If a significant moderating effect was identified, simple slope tests were performed to explore the nature of moderation further. Statistical significance was set at *P *< .05.

## Results

### Subjective ratings

**Figure 2.**
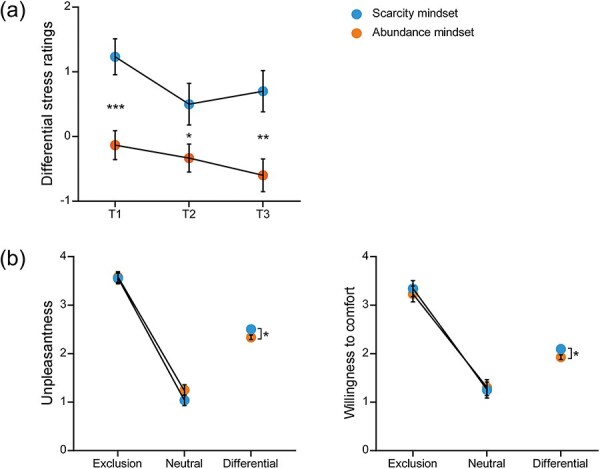
Subjective ratings and behavioral results. (a) Differential ratings of subjective stress under scarcity and abundance mindsets following each stage of the game. (b) Ratings of unpleasantness (left) and willingness to comfort (right) in response to social exclusion and neutral pictures under both scarcity and abundance mindsets. Data are presented as mean ± SE. *P < .05; ** *P* < .01; *** *P* < .001.

For subjective ratings of stress, significant main effects were observed for Group [*F*_(1,58)_ = 10.97, *P* = .002, η_p_^2^ = 0.16] and Timepoint [*F*_(2,57)_ = 7.04, *P* = .002, η_p_^2^ = 0.20]. Participants reported higher stress ratings under the scarcity mindset compared to the abundance mindset (0.81 ± 0.25 vs −0.36 ± 0.25). Additionally, stress ratings were significantly higher at T1 than at T2 (0.55 ± 0.18 vs 0.08 ± 0.19, *P* = .002) and T3 (0.55 ± 0.18 vs 0.05 ± 0.20, *P* = .005). The Group × Timepoint interaction was also significant [*F*_(2,57)_ = 3.36, *P* = .042, η_p_^2^ = 0.11]. Post hoc comparisons revealed that stress ratings at all three timepoints were significantly higher under the scarcity mindset compared to the abundance mindset (T1: 1.23 ± 0.25 vs −0.13 ± 0.25, *P* < .001; T2: 0.50 ± 0.27 vs −0.33 ± 0.27, *P* = .035; T3: 0.70 ± 0.29 vs −0.60 ± 0.29, *P* = .002) (see Fig. 2a).

No significant differences were found in the dimensions of confidence (*P* = .364), motivation (*P* = .115), or excitement (*P* = .100) between the scarcity and abundance mindset groups.

### Behavioral results

The effect size within the two LMMs was assessed using the MuMIn package (Bartoń 2023). Cohen’s *f*^2^ was calculated to quantify the relative explanatory power of fixed effects.

#### Unpleasantness

The Bayesian Information Criterion (BIC) for the LMM was 12892.26. The marginal *R*^2^ was calculated as 0.493, indicating that the fixed effects accounted for approximately 49.3% of the variance in the response variable. Consequently, the derived Cohen’s *f*^2^ value was approximately 0.973, suggesting a large effect size according to Cohen’s guidelines (Cohen 2013). Detailed results of the fixed effects are presented in Table 2. A significant main effect of Picture was observed, with higher unpleasantness ratings for social exclusion pictures compared to socially neutral pictures (3.56 ± 0.08 vs 1.14 ± 0.08, *P* < .001). Additionally, the Group × Picture interaction was significant. Simple effects analysis revealed that unpleasantness ratings for social exclusion pictures were significantly higher than those for socially neutral pictures under both mindsets (scarcity: 3.55 ± 0.11 vs 1.04 ± 0.11, *P* < .001; abundance: 3.58 ± 0.11 vs 1.25 ± 0.11, *P* < .001). However, the difference in ratings (social exclusion−social neutral) was significantly greater under the scarcity mindset compared to the abundance mindset (2.50 ± 0.05 vs 2.34 ± 0.05, *P* = .014) (see Fig. 2b).

**Table 2.** The detailed results for all fixed effects in two LMMs

Fixed effects	Est.	SE	*t*	*P*
Unpleasantness				
Intercept	5.92	0.13	46.36	<.001***
Group	0.13	0.18	0.71	.481
Picture	−2.34	0.05	−49.07	<.001***
Group × Picture	−0.17	0.07	−2.46	.014*
Willingness to comfort				
Intercept	5.16	0.18	28.89	<.001***
Group	0.28	0.25	1.10	.274
Picture	−1.93	0.05	−39.22	<.001***
Group × Picture	−0.17	0.07	−2.38	.017*

*P < .05;

***P < .001.

Regarding the random effects structure, the variance of the random intercepts for participants was estimated at 0.32 (SD = 0.57), indicating a significant variation in the baseline levels of the response variable among participants. The residual variance, which accounts for the unexplained variability within participants, was estimated to be 1.19 (SD = 1.10).

#### Willingness to comfort

The BIC for the LMM was 13 201.42. The marginal *R*^2^ was calculated as 0.331, indicating that the fixed effects accounted for approximately 33.1% of the variance in the response variable. Consequently, the derived Cohen’s *f*^2^ value was approximately 0.496, suggesting a large effect size. Detailed results of the fixed effects are presented in Table 2. A significant main effect of Picture was observed, with higher willingness to comfort ratings for social exclusion pictures compared to socially neutral pictures (3.29 ± 0.12 vs 1.28 ± 0.12, *P* < .001). The Group × Picture interaction was also significant. Simple effects analysis revealed that willingness to comfort ratings were significantly higher for social exclusion pictures than for socially neutral pictures under both mindsets (scarcity: 3.34 ± 0.16 vs 1.25 ± 0.16, *P* < .001; abundance: 3.23 ± 0.16 vs 1.30 ± 0.16, *P* < .001). Furthermore, the difference in ratings (social exclusion−social neutral) was significantly greater under the scarcity mindset compared to the abundance mindset (2.09 ± 0.05 vs 1.93 ± 0.05, *P* = .017) (see Fig. 2b).

Regarding the random effects structure, the variance of the random intercepts for participants was estimated to be 0.78 (SD = 0.88), indicating a significant variation in the baseline levels of the response variable among participants. The residual variance, which reflects the unexplained variability within participants, was estimated at 1.27 (SD = 1.13).

### Event-related potential results

#### N2

**Figure 3.**
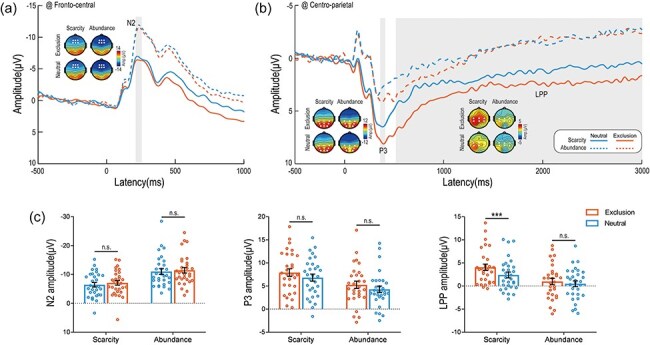
ERP results. (a) Grand-averaged ERP waveforms recorded at fronto-central electrodes in response to social exclusion and neutral pictures under scarcity and abundance mindsets. Scalp topographies illustrate the distributions of N2 amplitudes for each condition. (b) Grand-averaged ERP waveforms obtained from centro-parietal electrodes in response to social exclusion and neutral pictures under scarcity and abundance mindsets. Scalp topographies depict the distributions of P3 and LPP amplitudes for each condition. (c) Bar charts displaying the N2, P3, and LPP components. Significant differences in ERP waveforms are indicated by gray shaded squares. Electrodes used for estimating ERP amplitudes are marked with white points on their respective scalp maps. Data are presented as mean ± SEM. n.s.: *P* > .05; *** *P* < .001.

A significant main effect of Group was observed [*F*_(1,58)_ = 13.19, *P* = .001, η_p_^2^ = 0.19], indicating that smaller N2 amplitudes were elicited under the scarcity mindset compared to the abundance mindset (−6.76 ± 0.87 μV vs −11.24 ± 0.87 μV). Additionally, a significant main effect of Picture was found [*F*_(1,58)_ = 5.20, *P* = .026, η_p_^2^ = 0.08] with smaller N2 amplitudes elicited by social exclusion pictures compared to neutral pictures (−8.72 ± 0.63 μV vs −9.27 ± 0.63 μV). The interaction effect was not significant [*F*_(1,58)_ = 0.18, *P* = .675, η_p_^2^ < 0.01] (see Fig. 3a and c).

#### P3

A significant main effect of Group was found [*F*_(1,58)_ = 5.90, *P* = .018, η_p_^2^ = 0.09], indicating that greater P3 amplitudes were elicited under the scarcity mindset compared to the abundance mindset (7.36 ± 0.76 μV vs 4.76 ± 0.76 μV). Additionally, a significant main effect of Picture was observed [*F*_(1,58)_ = 27.61, *P* < .001, η_p_^2^ = 0.32] with greater P3 amplitudes elicited by social exclusion pictures compared to neutral pictures (6.58 ± 0.57 μV vs 5.54 ± 0.52 μV). The interaction effect was not significant [*F*_(1,58)_ = 0.10, *P* = .756, η_p_^2^ < 0.01] (see Fig. 3b and c).

### Late positive potential

A significant main effect of Group was found [*F*_(1,58)_ = 7.63, *P* = .008, η_p_^2^ = 0.12] indicating that greater LPP amplitudes were elicited under the scarcity mindset compared to the abundance mindset (3.22 ± 0.64 μV vs 0.74 ± 0.64 μV). Additionally, a significant main effect of Picture was observed [*F*_(1,58)_ = 22.93, *P* < .001, η_p_^2^ = 0.28], with greater LPP amplitudes elicited by social exclusion pictures compared to neutral pictures (2.52 ± 0.48 μV vs 1.44 ± 0.44 μV). Importantly, a significant Group × Picture interaction was identified [*F*_(1,58)_ =6.61, *P* = .013, η_p_^2^ = 0.10]. Post hoc comparisons revealed that under the scarcity mindset, social exclusion pictures elicited significantly greater LPP amplitudes than social neutral pictures (4.06 ± 0.69 μV vs 2.39 ± 0.63 μV, *P* < .001). In contrast, when participants were under the abundance mindset, LPP amplitudes did not differ significantly between social exclusion and neutral pictures (social exclusion: 0.99 ± 0.69 μV; social neutral: 0.49 ± 0.63 μV, *P* = .122) (see Fig. 3b and c).

### Brain–behavior relationship

We investigated the role of empathy traits in the relationship between neural and behavioral responses, specifically focusing on the LPP, a key ERP component linked to the observation of social exclusion stimuli (He et al. 2020, Wang et al. 2021, Zhao et al. 2021). In our moderation models, the LPP amplitude (calculated as social exclusion minus social neutral) served as the independent variable (X), the rating of willingness to comfort (calculated as social exclusion minus social neutral) was the dependent variable (Y), and the affective empathy trait score acted as the moderator variable (Mo).

**Figure 4.**
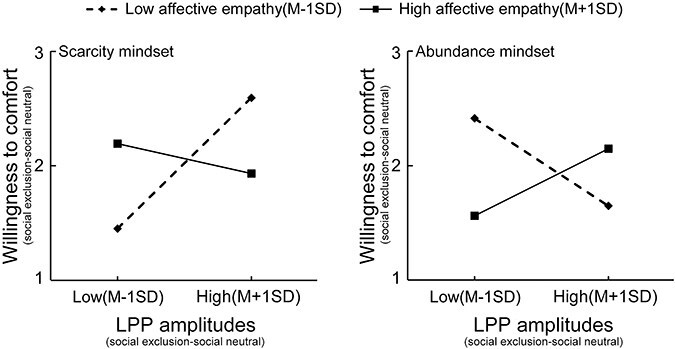
Simple slope charts. Sores of affective empathy trait moderated the link between LPP amplitudes (social exclusion–social neutral) and ratings of willingness to comfort (social exclusion–social neutral) under scarcity mindset, but not under abundance mindset, with low and high values of each moderator correspond to +1/−1 SD from the mean.

The analysis revealed that under the scarcity mindset, the moderation model was statistically significant (*R*^2^ = 0.29, *P* = .030). The regression equation was Y = − 0.48 + 1.44*X + 0.07*Mo − 0.04*X × Mo. Notably, the regression coefficients for the independent variable X (*Β* = 1.44, *t* = 2.75, *P* = .011, 95% CI = [0.364, 2.507]) and the interaction X × Mo (*Β* = − 0.04, *t* = − 2.55, *P* = .017, 95% CI = [−0.072, −0.008]) were both significant. Furthermore, the additional explanatory power gained by including the interaction term was also significant (∆R^2^ = 0.18, *P* = .017). Simple slope tests indicated that for individuals with low affective empathy traits, LPP amplitudes significantly predicted the ratings of willingness to comfort (*Β* = 0.33, *t* = 2.82, *P* = .009, 95% CI = [0.089, 0.572]). In contrast, for individuals with high affective empathy traits, LPP amplitudes did not significantly predict the ratings of willingness to comfort (*Β* = − 0.075, *t* = − 0.65, *P* = .521, 95% CI = [−0.314, 0.163]). However, under the abundance mindset, the moderation model did not reach statistical significance (*R*^2^ = 0.11, *P* = .384) (see Fig. 4 and Supplementary material S3 for further details).

## Discussion

This study investigated the impact of a scarcity mindset on individuals’ empathy for social pain and prosocial intentions toward others. We examined the differences in self-reported measures, neural responses, and the relationship with personality traits related to empathy between groups with scarcity and abundance mindsets. Behaviorally, the scarcity mindset group demonstrated greater differentiation in their ratings of unpleasantness and willingness to comfort between social exclusion and neutral stimuli compared to the abundance mindset group. On the neural level, we observed differences in early N2 and late P3 responses between scarcity and abundance mindsets for both social exclusion and neutral stimuli. Notably, LPP amplitudes in response to social exclusion and neutral stimuli varied significantly under the scarcity mindset, whereas they were comparable under the abundance mindset. Furthermore, when participants experienced a scarcity mindset, their affective empathy emerged as a crucial moderator in the relationship between LPP amplitudes and willingness to comfort ratings.

The stage-game paradigm was utilized to manipulate participants’ feelings of scarcity or abundance. The effectiveness of this manipulation was assessed through subjective ratings collected before the study and after each stage of the game, with a particular emphasis on stress ratings, as highlighted in previous studies (Huijsmans et al. 2019, Li et al. 2023). In this study, we successfully induced a scarcity mindset. Following the manipulation, individuals in the scarcity mindset group consistently reported higher levels of stress throughout the experiment than those in the abundance mindset group.

Evaluating the unpleasantness level of target individuals is a common indicator in the analysis of social exclusion images (Zhao et al. 2021, Mo et al. 2023). This indicator was chosen to assess empathy for social pain, while the willingness to comfort the target individual was included to measure prosocial intentions. Our findings for these two indicators were consistent: both groups reported significantly higher ratings for social exclusion images than for socially neutral ones, with the differences being more pronounced in the scarcity mindset group than in the abundance mindset group. It is important to note that while the unpleasantness ratings for social exclusion images were marginally lower under the scarcity mindset than under the abundance mindset, the scarcity mindset amplified the distinction between the two types of images. This observation aligns with the findings of Kraus et al. (2010), which demonstrated that individuals from lower social classes exhibited heightened empathic accuracy regarding others’ social emotions. Furthermore, a scarcity mindset is considered a form of psychosocial stress (Huijsmans et al. 2019). Our results are consistent with previous research indicating that psychosocial stress can lead to increased self-reported empathic responses (Gonzalez-Liencres et al. 2016) and prosocial behaviors (von Dawans et al. 2012, Tomova et al. 2017). This supports the “Tend and Befriend” model, which suggests that stress fosters affiliative behaviors, prompting individuals to seek protection and support from others (Taylor 2006, von Dawans et al. 2012, Azulay et al. 2022). These findings reveal that a scarcity mindset enhances both empathy for social pain and the intention to engage in prosocial behaviors by increasing sensitivity to others’ social suffering.

Correspondingly, the self-reported empathic ratings were supported by electrophysiological evidence, which revealed increased amplitudes of the LPP component under a scarcity mindset compared with an abundance mindset. Owing to the complexity of social exclusion and neutral images, the selected time window for LPP amplitudes was relatively long (Zhao et al. 2021), diverging from standard ERP analysis practices. Consistent with previous research, we observed a significant LPP component triggered by the passive viewing of social exclusion stimuli (He et al. 2020, Zhao et al. 2021). While the LPP component is typically associated with top-down attentional processing (Hajcak et al. 2010, Hajcak and Foti 2020), it also reflects cognitive engagement with emotionally salient stimuli, including the cognitive appraisal of others’ emotional states (Myruski et al. 2019). Our findings indicate that participants under a scarcity mindset exhibited enhanced LPP amplitudes in response to social exclusion, suggesting an increased cognitive effort in evaluating the emotional states of others. This supports the idea that individuals experiencing scarcity allocate more cognitive resources to appraising social stimuli (Li et al. 2023).

The LPP component is recognized as a reliable electrophysiological indicator of empathy for pain (Coll 2018), reflecting the top-down cognitive processes involved in evaluating others’ affective experiences (Fan and Han 2008). Consequently, the effect of a scarcity mindset on empathic neural responses to social pain primarily manifests in the top-down cognitive processing of ostracized victims’ emotional states. When observers encounter someone experiencing social pain, they may not immediately grasp the intensity of their suffering. Instead, they rely on prolonged observation and appraisal of the affective states to understand the individual’s “pain”. For individuals under a scarcity mindset, cognitive capacity may be diminished due to the constraints of scarcity (Mani et al. 2013). As a result, when instructed to focus on target individuals, they are likely to allocate limited mental resources to appraise the target’s emotional states, leading to a more nuanced evaluation. On the other hand, the LPP component over the central-parietal region is associated with top-down emotion regulation processes (Myruski et al. 2019, Hajcak and Foti 2020). During emotion regulation, LPP amplitudes typically decrease (He et al. 2020; Li et al. 2022). In addition to the mental resources dedicated to evaluation, participants under an abundance mindset may attempt to regulate their emotional responses to the affective states of excluded victims, albeit with limited mental resources. Consequently, the LPP amplitudes under an abundance mindset exhibited a negative shift relative to those under a scarcity mindset. In contrast to the scarcity mindset’s influence on the bottom-up and top-down stages of empathy for physical pain, a scarcity mindset selectively affected the top-down attentional processing stage during empathy for social pain. Empathy for social pain is less automatic than empathy for physical pain (Masten et al. 2010), making it more challenging for individuals to quickly discern whether others are experiencing social pain and to share their unpleasant emotions within a short timeframe.

In summary, a scarcity mindset enhanced the cognitive processing stage of empathy for social pain and the associated comforting intentions. It is important to note that this effect contrasts with previous findings indicating that individuals’ empathy for physical pain (Li et al. 2023) and their intentions to share were diminished under a scarcity mindset (Aarøe and Petersen 2013, Cui et al. 2022, 2023). According to the self-regulatory model of resource scarcity, individuals typically navigate two key psychological pathways in response to resource scarcity: the scarcity-reduction route, which motivates individuals to acquire resources to directly address the resource gap, and the control-restoration route, which encourages individuals to seek security in other domains (Cannon et al. 2019). Those who exhibit reduced empathy for physical pain and diminished intentions to share under a scarcity mindset may follow the scarcity-reduction route. In these situations, resources are often overvalued (Shah et al. 2012), leading individuals to focus on acquiring resources for themselves while neglecting the physiological needs of others. This focus can result in indifference to others’ suffering (e.g. actual physical pain) and a reluctance to share resources. Conversely, individuals in our study who had a scarcity mindset may have been following the control-restoration route. Despite experiencing resource scarcity, these individuals remain attuned to the emotions of others and accurately identify those emotions (Kraus et al. 2010, 2012). They are likely to establish affective bonds through empathy to find a sense of security. As a result, they are more inclined to be concerned about others’ social and emotional needs and to respond to the suffering caused by social interactions (i.e. social pain), demonstrating comforting intentions toward excluded victims. The different pathways individuals take in response to scarcity lead to varying emotional reactions to the suffering of others and differing prosocial intentions. The choice of route may be influenced by various contextual factors. However, these interpretations remain speculative, and further research is needed to explore the underlying mechanisms that contribute to these distinctions in greater detail.

Furthermore, individuals under a scarcity mindset exhibited smaller N2 amplitudes and larger P3 amplitudes than those under an abundance mindset, regardless of the stimulus type. The early N2 component is associated with the automatic emotional contagion stage during empathy processing (Fan and Han 2008, Cui et al. 2016). The reduced negative deflection in N2 amplitudes observed under a scarcity mindset suggests that individuals may struggle to capture automatic neural responses to complex emotional information, leading to a decreased sensitivity to others’ emotions during the early processing stage. On the other hand, the P3 component reflects late cognitive processing of emotional stimuli (Fan and Han 2008, Wei et al. 2023). Previous research has indicated that challenging contexts can enhance P3 responses, which suggests increased cognitive engagement (Wu et al. 2020). Similarly, individuals with a scarcity mindset may encounter cognitive challenges due to resource limitations (Mani et al. 2013, Shah et al. 2015). Consequently, they may demonstrate more positive-going P3 amplitudes compared to those in an abundance mindset, indicating heightened cognitive processing in response to emotional stimuli.

Interestingly, we found evidence of a moderated effect of affective empathy traits on the relationship between neural responses associated with empathy for social pain and behavioral judgments regarding prosocial intentions within the scarcity mindset group. In individuals with low affective empathy scores who experienced a scarcity mindset, greater LPP amplitudes were predictive of a higher willingness to comfort others. This relationship was not observed among those with high affective empathy scores. A scarcity mindset may amplify the facilitating effect of empathic neural responses on subjective prosocial intentions among individuals with low affective empathy. While viewing empathy as a limited resource may diminish empathic responses, engaging in effortful cognitive processing can enhance empathy and prosocial behavior toward others (Hasson et al. 2022). Consequently, individuals with low affective empathy traits may invest greater effort in utilizing mental resources to process and evaluate the affective states of others, resulting in higher prosocial intentions. In contrast, individuals with high affective empathy traits may find it relatively easier to empathize with excluded victims (Tao et al. 2020), even when faced with the mental resource challenges posed by a scarcity mindset. Therefore, when individuals have a scarcity mindset, those with low affective empathy traits may still demonstrate prosocial intentions toward others who are socially excluded, but they may need to mobilize more mental resources compared to their high affective empathy counterparts.

Several limitations should be acknowledged in this study. First, the stimulus materials included only images depicting social exclusion and neutral scenarios without including social inclusion images. As the perception of neutral stimuli can sometimes be biased toward negativity rather than remaining entirely neutral (Tae et al. 2020), future research should incorporate social inclusion images to provide a more comprehensive understanding of empathy in complex social interactions. Second, although our sample size met the standards for achieving sufficient statistical power, it may not have fully captured the entire spectrum of empathy levels present in the population. A larger sample size would not only enhance the reliability of the statistical outcomes but also provide a better representation of individuals with varying levels of empathy. Consequently, the results of this study should be considered preliminary. Future studies with larger sample sizes could further investigate how individuals with high versus low empathy are influenced by a scarcity mindset and how they respond to social exclusion scenarios.

## Conclusions

This study revealed that a scarcity mindset enhanced the later cognitive appraisal stage of empathy for social pain, as indicated by the unpleasantness ratings and the LPP component. Additionally, it facilitated prosocial intentions toward excluded victims, as measured by the willingness to comfort ratings. Furthermore, the role of affective empathy traits emerged as pivotal in shaping the relationship between empathy and prosocial behavior in the context of a scarcity mindset. These findings enrich our understanding of the neural mechanisms underlying empathy for social pain and highlight the significant impact of empathy traits, thereby providing deeper insights into individuals’ social behaviors when resources are limited.

## Supplementary Material

nsaf015_Supp

## Data Availability

The data associated with this study can be found at https://pan.baidu.com/s/15Ad9AR2jTyCEvKpu6-js3A?pwd=2023.
