# Supplementary material for: Scarcity mindset facilitates empathy for social pain and prosocial intention: behavioral and neural evidences
Source: Soc Cogn Affect Neurosci. 2025 Jan 28;20(1):nsaf015. doi: 10.1093/scan/nsaf015 (PMC11803631; doi:10.1093/scan/nsaf015)
Supplement: nsaf015_Supp [file nsaf015_supp.zip › scan-24-154-File007.docx]

**Supplementary materials**

**S1 Stage-game paradigm**

The stage-game paradigm includes three cognitive/perceptual tasks, namely, dot comparison, shape matching, and dot counting. The difficulty of these games is comparable. Specifically, for the dot comparison task, participants were instructed to determine which box had more dots. In fact, both boxes always contained an equal number of dots, with the number varying between 30 and 40. In the shape matching task, participants were instructed to judge whether the two presented shapes together could form a perfect circle. In fact, none of the shapes met the requirement for forming a perfect circle. And in the dot counting task, participants were instructed to estimate whether the number of dots displayed on the screen was more or less than the specified target number (32 or 38). The actual number of dots varied between 30 and 40 (Huijsmans et al., 2019).

**S2 Questions for subjective ratings**

All ratings were performed on 9-point Likert scales.

1. When you got 1 (or 10) token(s) at the beginning of this game, to what extent did you feel stressful? (1=very stress-free and 9=very stressful)
2. When you got 1 (or 10) token(s) at the beginning of a game, to what extent did you feel confident? (1=very unconfident and 9=very confident)
3. When you got 1(or 10) token(s) at the beginning of a game, to what extent did you feel motivated? (1=very unmotivated and 9=very motivated)
4. When you got 1 (or 10) token(s) at the beginning of a game, to what extent did you feel excited? (1=very unexcited and 9=very excited)

**S3 Moderation Analysis Results under scarcity mindset**

We used SPSS's PROCESS macro for moderation analysis (Model 1), where empathy scores served as a continuous moderator. We selected values at +1SD, M, and −1SD as conditioning values to explore the moderating effects of empathy traits. The output actually calculated the effect at three levels of the moderator (+1SD, M, and −1SD). To keep the concise of the results, we present the +1SD, and −1SD in the main text as the previous studies did.

| **Moderation Analysis Results under scarcity mindset** | | | | | |
| --- | --- | --- | --- | --- | --- |
| Variable | *Coefficient* | *SE* | *t* | *p* | 95% CI |
| constant | 2.04 | 0.14 | 14.11 | < 0.001 | [1.744, 2.339] |
| LPP amplitudes | 0.13 | 0.09 | 1.50 | 0.147 | [-0.048, 0.303] |
| affective empathy | 0.00 | 0.03 | 0.12 | 0.903 | [-0.062, 0.070] |
| interaction | -0.04 | 0.02 | -2.55 | 0.017 | [-0.072, -0.008] |

Note: All coefficients are unstandardized. The same applies below.

| **Conditional Effects at Different Levels of Affective Empathy** | | | | | | |
| --- | --- | --- | --- | --- | --- | --- |
|  | *M* | *B* | *SE* | *t* | *p* | 95% CI |
| Low | 25.20 ± 2.39 | 0.33 | 0.12 | 2.82 | 0.009 | [0.089, 0.572] |
| Middle | 32.80 ± 2.95 | 0.13 | 0.09 | 1.50 | 0.147 | [-0.048, 0.303] |
| High | 39.80 ± 2.49 | -0.08 | 0.12 | -0.65 | 0.521 | [-0.314, 0.163] |


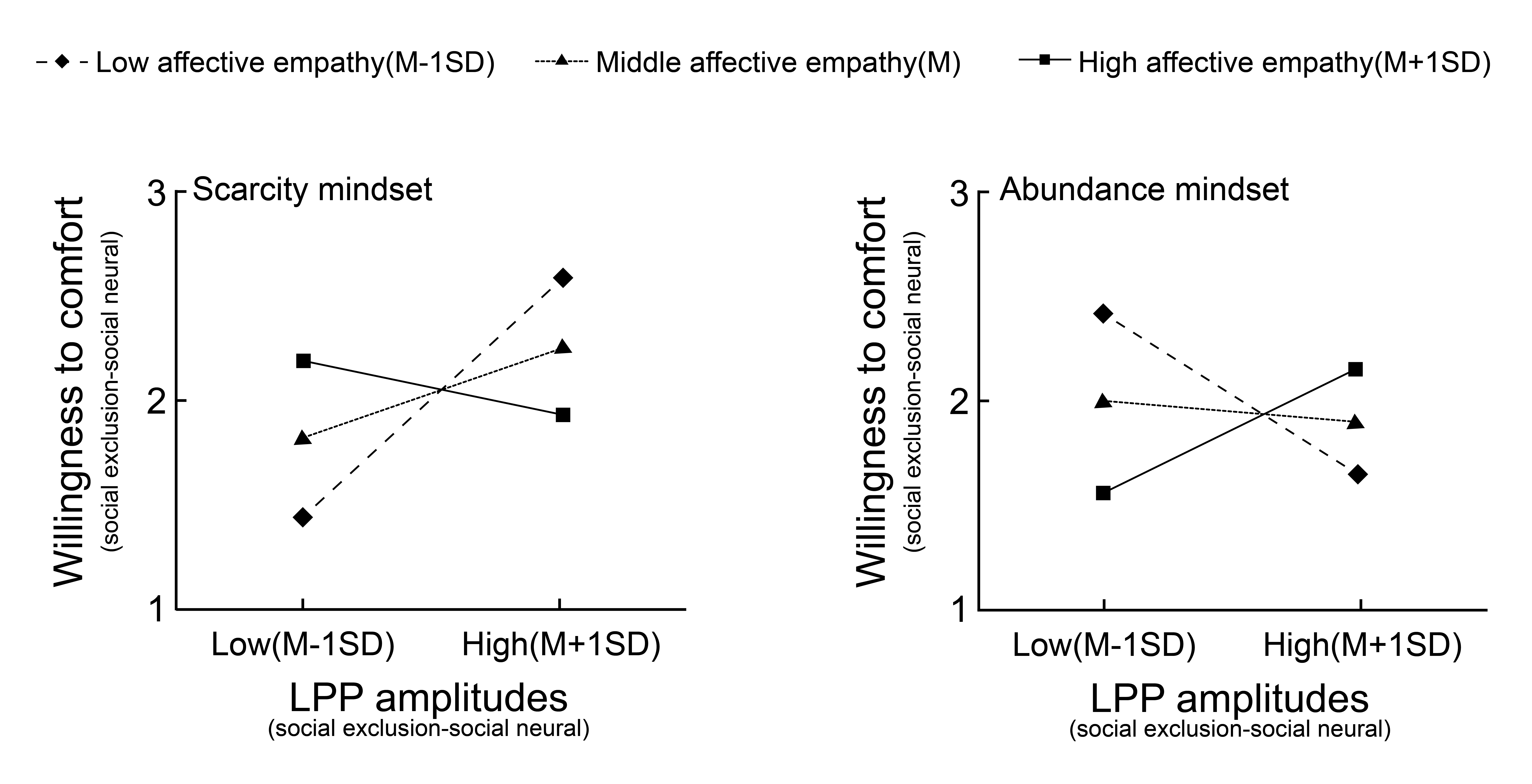


**Figure S1.** **Simple slope charts.** Sores of affective empathy trait moderated the link between LPP amplitudes (social exclusion–social neutral) and ratings of willingness to comfort (social exclusion–social neutral) under scarcity mindset, but not under abundance mindset. Low, middle and high values of each moderator correspond to +1 SD, mean, and−1 SD from their means.

**References**

Huijsmans, I., Ma, I., Micheli, L., Civai, C., Stallen, M., & Sanfey, A. G. (2019). A scarcity mindset alters neural processing underlying consumer decision making. *Proc Natl Acad Sci U S A*, *116*(24), 11699-11704. https://doi.org/10.1073/pnas.1818572116
